# Clinical and psychopathological features of very late onset of schizophrenia-like psychosis

**DOI:** 10.1192/j.eurpsy.2024.705

**Published:** 2024-08-27

**Authors:** V. Pochueva, I. Kolykhalov

**Affiliations:** ^1^FSBSI Mental Health Research Center, Moscow, Russian Federation

## Abstract

**Introduction:**

Very late onset schizophrenia-like psychosis takes the 3^rd^ place among late-life psychosis, after dementia and affective disorders associated psychosis. It’s still unknown the real place of this psychosis.

**Objectives:**

to investigate the clinical and psychopathological features and short-terms outcomes of late-onset schizophrenia and schizophrenia-like psychosis

**Methods:**

45 patients, mean age 70,6 ±8,70 years, median age of manifestation psychosis - 68 [61; 75] years with late-onset schizophrenia (n=19, 42,2%), late-onset schizoaffective disorder (n=9, 20%), late-onset delusional disorder (n=7, 15,5%) and late-onset organic schizophrenia-like disorder (n=10, 22,3%) underwent clinical examination. Psychopathological, psychometric (PANSS, HAMD, CDSS, MoCA) and statistical methods were applied.

**Results:**

3 clinical groups were allocated. The 1^st^ group included 15 patients (33%) and was characterized with severe polymorphic psychotic symptoms, included catatonic and paraphrenic signs with mental disorganization. They had the highest score of PANSS (105,46±17,99, p=0,002) and the lowest score of MoCA (14,2±2,16, p=0,05) in compare with 2^nd^ and 3^rd^ groups. They also had symptoms of depression (CDSS 6,28±5,29), compared with the 3^rd^ group (HAMD 21,00±5,92, p=0,05). In short-terms outcomes was formed negative symptoms and cognitive impairment with decreasing social and daily activity. The 2^nd^ group (22 cases, 49%) included patients with prevalence of delusions of persecution, more rare auditory hallucinations and more often acoasms. They had medium score PANSS (90,22±16,79), with minimal cognitive declare (MoCA average score 20,33±4,27). The short-term outcomes were characterized with formation of residual positive and negative symptoms, that impact on daily and social activity. The 3^rd^ group included 8 patients (8%) with prevalence of delusion symptoms, such as misidentification, persecutory and reference delusions, which were mood-congruent. They had medium PANSS score (89,75±18,90) with more severe depressive symptoms by HAMD scale in compare with 2^nd^ group (22,00±10,00, p=0,07) and minimal cognitive declare (MoCA average score 25,00±1,00, p=0,05) in compare with 1^st^ group. This group was characterized with high level of reduction of productive symptoms and restoration of premorbid social and daily activity in short-term outcomes.

**Conclusions:**

features of clinical characteristics, including the nature and severity of cognitive impairment at the onset of disease, are significant for prognosis and outcomes of disease. The data obtained could be served for the development of personalized therapeutic approaches that take into account the syndromic features and course of late-onset psychosis.

**Disclosure of Interest:**

None Declared

